# An Indirect Organogenesis-Based Citrus Transformation System Monitored Using Visible Reporter Markers

**DOI:** 10.3390/plants15203141

**Published:** 2026-10-14

**Authors:** Sameena Ejaz Tanwir, Tao Jiang, Anandi Karn, Carlos D. Messina, Heqiang Huo

**Affiliations:** 1 Crop Transformation Center, Department of Horticultural Sciences, Institute of Food And Agricultural Sciences, University of Florida, Gainesville, FL 32611, USA; tanwir.s@ufl.edu (S.E.T.); taojiang@ufl.edu (T.J.); anandikarn@ufl.edu (A.K.); cmessina@ufl.edu (C.D.M.); 2 Mid-Florida Research & Education Center, Institute of Food And Agricultural Sciences, University of Florida, Apopka, FL 32703, USA

**Keywords:** citrus, genetic transformation, indirect organogenesis, visible reporter, *ROSEA1*, *RUBY*

## Abstract

Genetic transformation of citrus is essential for functional genomics and trait improvement, yet remains limited by prolonged regeneration cycles, genotype-dependent responses, and inefficient screening of transformed tissues. Here, we established an *Agrobacterium*-mediated indirect organogenesis-based transformation system for Carrizo citrange and evaluated two visible reporter systems, the anthocyanin regulator *ROSEA1* and the betalain biosynthetic cassette *RUBY*, for monitoring transformed tissues throughout regeneration. The regeneration protocol enabled visible callus initiation within 7 days following a 5-day pre-culture treatment and recovery of PCR-confirmed transgenic plantlets within 5 months. Both visible reporters were compatible with callus development and shoot regeneration throughout the indirect organogenesis workflow. During callus proliferation, visible pigmentation closely corresponded with GFP fluorescence, enabling rapid, non-destructive identification of transformed tissues. However, reporter performance diverged during shoot regeneration: *RUBY* maintained stable pigmentation throughout regeneration, whereas *ROSEA1*-associated pigmentation progressively declined despite continued GFP fluorescence. Consequently, *RUBY* showed an approximately 4.7-fold higher pigment-positive shoot fraction than *ROSEA1* at week 17, and the *RUBY*-positive fraction was similar to the GFP-positive fraction in the same treatment. Putative transgenic events were confirmed by PCR in independent lines of both constructs, together with GFP fluorescence in leaves and root tips. Together, these findings describe a practical indirect organogenesis-based transformation platform for citrus and demonstrate that both *ROSEA1* and *RUBY* are effective visual reporters during callus proliferation, whereas *RUBY* provides more reliable visual identification during shoot regeneration. *RUBY* is therefore recommended as the visible reporter of choice for citrus transformation extending beyond the callus stage. The workflow was established in Carrizo citrange, among the most readily transformed citrus genotypes, and extension to other varieties remains to be demonstrated.

## 1. Introduction

Citrus is one of the world’s most economically important fruit crops, with an annual production of approximately 161.8 million tons, and represents a major source of vitamin C, flavonoids, carotenoids, and other health-promoting phytochemicals [1,2]. However, global citrus production faces significant challenges from diseases like Huanglongbing (HLB), caused by *Candidatus Liberibacter asiaticus* (CLas), and citrus canker, caused by *Xanthomonas citri* subsp. *citri*. These diseases greatly reduce tree productivity, fruit quality, and orchard longevity worldwide [3,4,5]. These challenges demand the development of new citrus cultivars with improved disease resistance and key agronomic traits. Consequently, breeders have long worked to develop improved scion and rootstock cultivars through traditional breeding methods. However, these methods are time-consuming and labor-intensive and can take around 20 years from a cross to cultivar release [6]. Additionally, the complex genome structure and high heterozygosity of cultivated citrus [7], together with nucellar polyembryony, self-incompatibility, partial sterility, and a 5–8-year juvenile phase [8], make citrus breeding especially challenging. Therefore, there is a need to integrate modern precision breeding tools with conventional approaches to increase genetic gain per breeding cycle. Genetic transformation and genome editing address several of these constraints directly, and transgenic approaches targeting both citrus canker and huanglongbing have been developed and reviewed extensively [9].

However, the successful application of these technologies depends on efficient, reproducible plant regeneration and transformation. Among gene delivery methods, *Agrobacterium*-mediated transformation is the most common in citrus because of its simplicity, broad applicability, and lack of requirement for protoplast culture or embryogenic cell suspensions [10,11]. Despite continuous improvements, transformation efficiency remains highly genotype-dependent and is often limited by prolonged in vitro culture, low recovery of transgenic events, and the occurrence of escapes and chimeric regenerants under antibiotic selection [12,13,14]. Most protocols have been optimized predominantly using juvenile seedling explants, especially epicotyl, cotyledon, and hypocotyl segments via direct organogenesis, whereas indirect organogenesis has been reported for fewer genotypes [12,15]. Indirect organogenesis involves a prolonged callus proliferation phase prior to shoot regeneration, providing an extended window during which transformed cells can proliferate and be selected, potentially improving the recovery of stable transgenic regenerants [16,17]. However, efficient and reproducible transformation systems based on indirect organogenesis remain limited in citrus.

Establishing an efficient regeneration and transformation system alone is insufficient for successful citrus transformation; reliable identification of transformed tissues throughout the prolonged regeneration process is equally important. Although selectable marker genes favorably select transformed cells, antibiotic selection alone does not always eliminate non-transformed tissues, leading to escapes and chimeric regenerants during prolonged culture [11,18]. Therefore, reporter genes are routinely incorporated into transformation vectors to facilitate identification and recovery of transgenic events. The most widely used reporters are protein-based, including β-glucuronidase (*GUS*), luciferase (*LUC*), and green fluorescent protein (GFP), each with inherent limitations for long-term regeneration studies. *GUS* requires destructive histochemical staining, precluding repeated assessment of the same explant [19]. *LUC* depends on exogenous substrate application and specialized imaging. GFP enables non-destructive, real-time visualization and has been widely used in citrus transformation [20], but its detection requires fluorescence imaging equipment and may be complicated by chlorophyll autofluorescence in regenerating green tissues [21,22,23]. These limitations have spurred the development of visible pigment reporters, which produce colored metabolites that can be detected directly under ambient light without exogenous substrates or specialized instrumentation [21,24].

Visible pigment reporters can be broadly divided into anthocyanin- and betalain-based systems, which differ fundamentally in their mechanisms of pigment production. *ROSEA1* is an R2R3-MYB transcription factor that activates the endogenous anthocyanin biosynthetic pathway through host regulatory networks [25], whereas the *RUBY* reporter encodes the three enzymes required for betalain biosynthesis as a single polycistronic cassette, enabling pigment production largely independent of endogenous anthocyanin regulatory pathways [21]. Both systems have been successfully applied for visual selection of transformed tissues in diverse plant species, including woody horticultural crops such as Mexican lime, bilberry, and apple [26,27,28]. However, these reporters have primarily been evaluated independently, and their performance during the successive stages of regeneration has not been systematically investigated in citrus, particularly in transformation systems based on indirect organogenesis.

In this study, an *Agrobacterium*-mediated transformation system based on indirect organogenesis was established for Carrizo citrange. Using a common GFP–NPTII backbone, the anthocyanin reporter *ROSEA1* (*ROS1*) and the betalain reporter *RUBY* were evaluated as visible markers throughout the transformation process, from callus induction to transgenic plant recovery. The objectives were twofold: (i) to establish an indirect organogenesis-based *Agrobacterium* transformation system for Carrizo citrange, including recovery of rooted plantlets without shoot-tip micrografting, and (ii) to determine whether a visible reporter can provide a practical, equipment-free complement to fluorescence-based screening in monitoring transformed tissue throughout that system, and if so which of the two reporters is better suited to the task. The second objective is not an addition to the first but its enabling component, since indirect organogenesis requires transformed tissue to be identified repeatedly across a prolonged culture period. No factorial optimisation of transformation parameters was undertaken, and the system was not compared side by side with a direct-organogenesis protocol; the efficiencies reported here are therefore presented as values achieved by this workflow rather than as evidence of superiority over existing citrus transformation systems.

## 2. Results

### 2.1. Establishment of an Indirect Organogenesis-Based Transformation System for Carrizo Citrange Using Visible Reporter Markers

**Figure 1:**
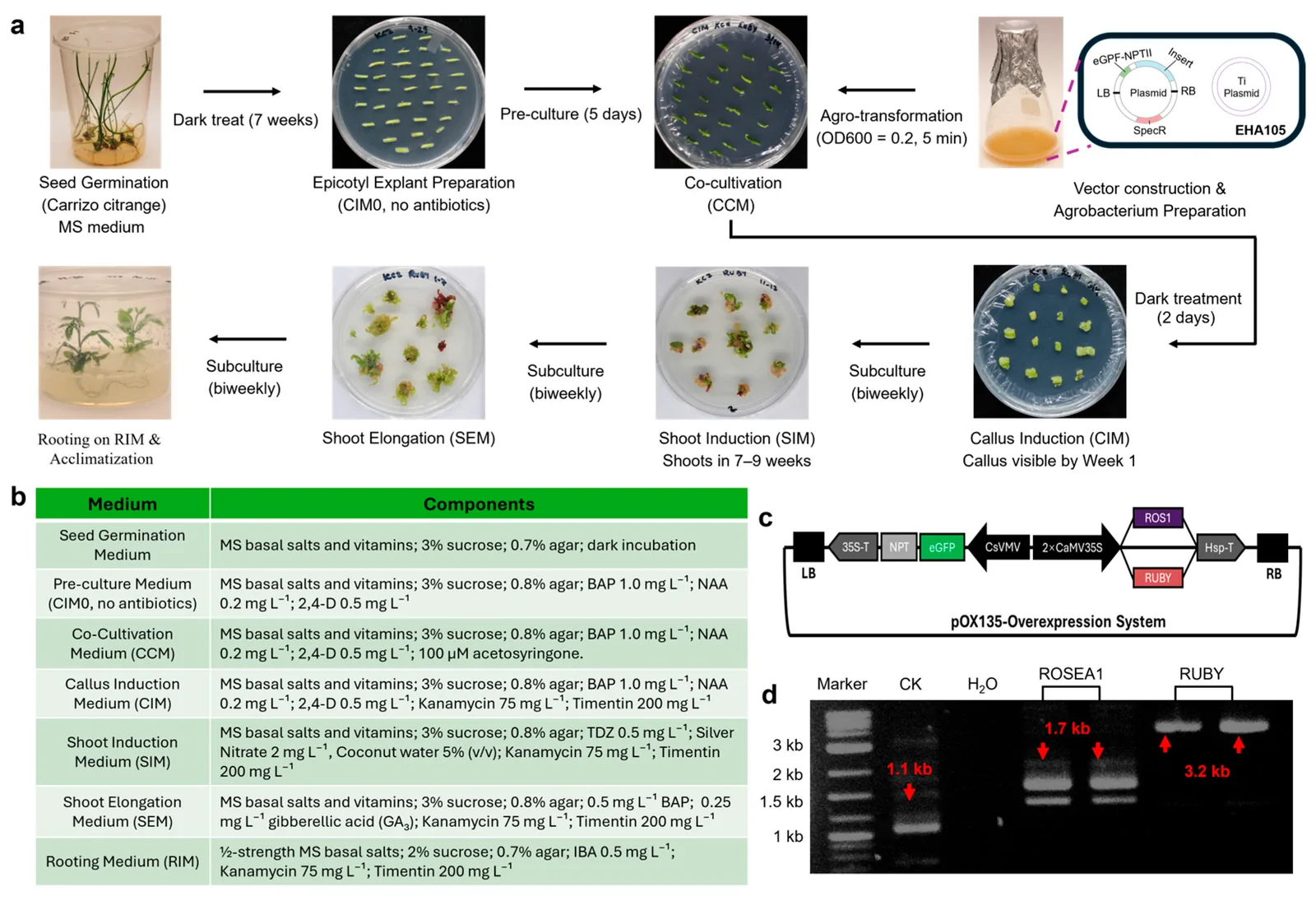
Establishment of an indirect organogenesis-based citrus transformation system using visible reporter markers. (**a**) Overview of the transformation and regeneration workflow, including seed germination, epicotyl preparation, pre-culture, *Agrobacterium* infection and co-cultivation, callus induction and selection, shoot regeneration and elongation, and rooting. (**b**) Composition of the culture media used at each stage of transformation and regeneration. CIM, callus induction medium; CCM, co-cultivation medium; SIM, shoot induction medium; SEM, shoot elongation medium; RIM, rooting medium. (**c**) Simplified T-DNA structures of the pOX135-based constructs. The control construct (CK) contains the CsVMV-driven eGFP-NPTII reporter-selection fusion cassette, whereas pOX135-*ROSEA1* (*ROS1*) and pOX135-*RUBY* additionally contain the visible reporter *ROSEA1* or *RUBY*, respectively, driven by the 2×CaMV 35S promoter and terminated by Hsp-T. (**d**) PCR verification of the assembled plasmids using cassette-flanking primers common to all three constructs. Lanes: M, GeneRuler 1 kb Plus DNA Ladder (Thermo Fisher); CK, unmodified pOX135 (expected 1.1 kb); H_2_O, no-template control; *ROS1*, pOX135-*ROSEA1* (expected 1.7 kb, two independent clones); *RUBY*, pOX135-*RUBY* (expected 3.2 kb, two independent clones). A minor secondary product of approximately 1.4 kb is generated by this primer pair and is visible in the *ROSEA1* lanes and, faintly, in the *RUBY* lanes; it does not correspond to any expected cassette product. Complete cassette integrity was confirmed by Sanger sequencing. Construct accessions are given in Section 4.4 and primer sequences in Table S1.

An *Agrobacterium*-mediated indirect organogenesis system was developed for Carrizo citrange to evaluate anthocyanin- and betalain-producing genes as visible reporters of transformation (Figure 1). Three constructs were assembled: the unmodified pOX135 vector as a control (CK), and pOX135-*ROSEA1* and pOX135-*RUBY*, in which anthocyanin and betalain biosynthesis, respectively, are driven by a 2×CaMV35S promoter (Figure 1c). All three contain the fused eGFP-NPTII cassette, so that each visible marker can be evaluated against GFP within a common vector background. Correct assembly of all constructs was confirmed by PCR prior to transformation (Figure 1d).

Etiolated epicotyl segments excised from dark-grown seedlings were used as explants and were carried through pre-culture, *Agrobacterium* inoculation, and co-cultivation, followed by callus induction, shoot induction, shoot elongation, and rooting under continued kanamycin selection (Figure 1a). Regenerated shoots were then transferred to a rooting medium containing Kanamycin 75 mg L^−1^ and Timentin 200 mg L^−1^. Media compositions for each stage are given in Figure 1b. Callus became visible within one week of transfer to selection medium, early shoot regeneration was detectable by week 5, with clearly developed adventitious shoots becoming prominent during weeks 7–9, and rooted plantlets were obtained by week 20.

### 2.2. Visible Reporter Expression Enables Non-Destructive Identification of Transformed Callus During Induction and Proliferation

**Figure 2:**
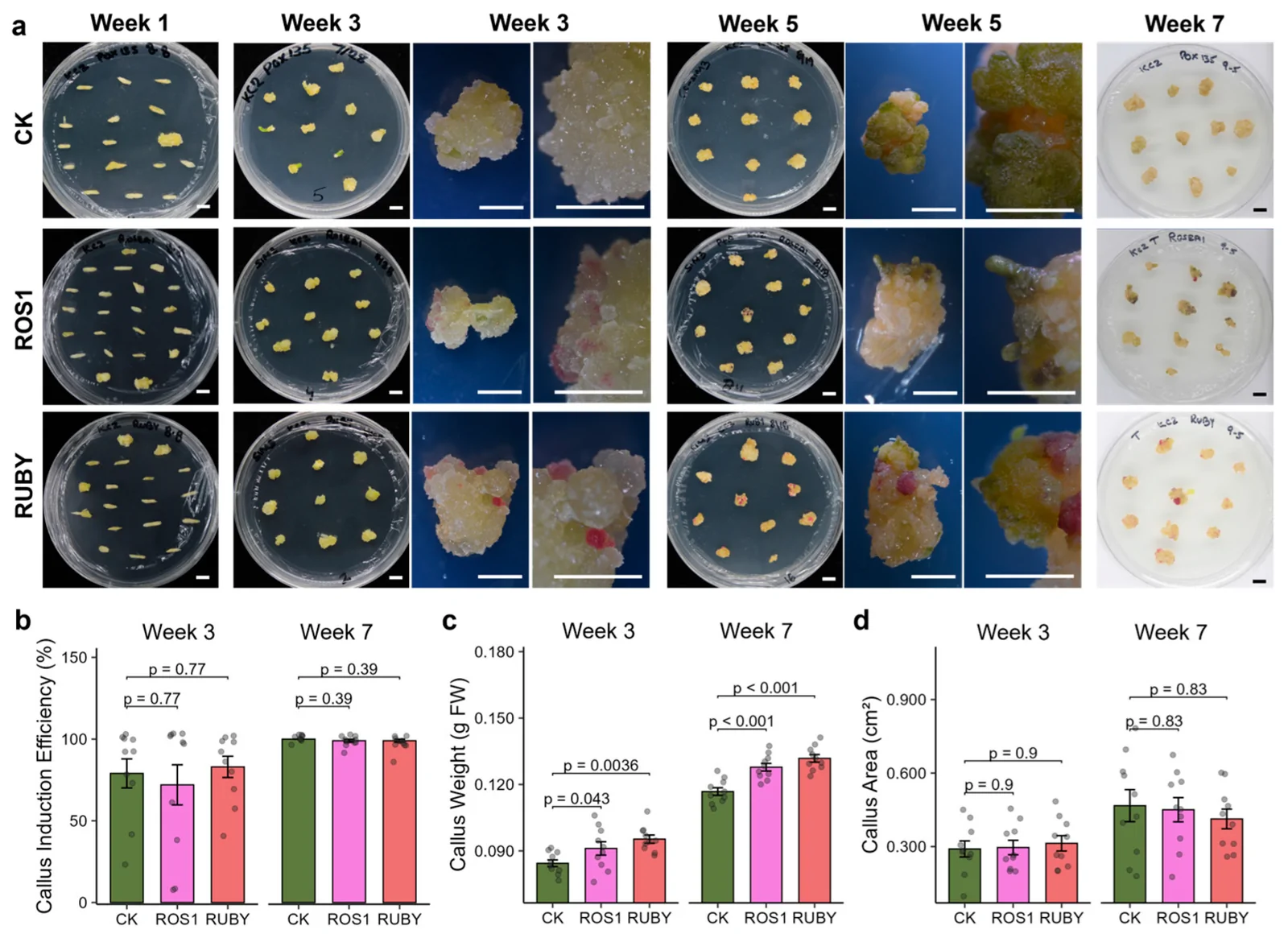
Visible markers *ROSEA1* and *RUBY* indicate callus growth status during transformation in citrus. (**a**) Representative phenotypes of citrus explants during *Agrobacterium*-mediated transformation under control (CK), *ROSEA1* (*ROS1*), and *RUBY* treatments at weeks 1, 3, 5, and 7. CK explants developed original calli without visible pigment accumulation, whereas *ROSEA1* and *RUBY* explants displayed pink-purple anthocyanin and betalain pigmentation, respectively. Representative Petri-dish overviews and close-up views of individual calli are shown. (**b**) Callus induction efficiency (%) (**c**) Callus weight (g fresh weight) (**d**) Callus area (cm^2^). CK (green), *ROSEA1* (purple), and *RUBY* (carmine) treatments at weeks 3 and 7. Data are presented as mean ± s.d. (*n* = 8–10 biological replicates, 50–60 explants per treatment, from at least three independent transformation experiments; individual replicate values are overlaid as points). Statistical significance was assessed using two-tailed Student’s *t*-tests (CK versus *ROSEA1*; CK versus *RUBY*). Scale bars, 1 cm (Petri dishes in (**a**)) and 5 mm (close-ups in (**a**)).

To determine whether constitutive visible reporter expression affected dedifferentiation, callus formation was compared among CK, *ROSEA1*, and *RUBY* explants over seven weeks on selective medium. Visible callus initiated at the cut surfaces of epicotyl explants within 7 days in all three treatments (Figure 2a). Control explants formed pale-yellow callus, whereas *ROSEA1* and *RUBY* tissues developed distinct pink-purple and carmine-red pigmentation, respectively, from week 3; pigmentation intensified by week 7, allowing reporter-expressing callus to be distinguished under ambient light (Figure 2a). Equivalent phenotypes were observed across independent biological replicates (Figures S1–S3).

Callus induction efficiency was unaffected by either reporter. Approximately 70–80% of explants formed callus by week 3 and close to 100% by week 7, with no significant difference between either marker treatment or CK at either timepoint (Figure 2b; week 3, *p* = 0.77; week 7, *p* = 0.39). In contrast, callus fresh weight was significantly higher in both reporter treatments than in CK. At week 3, *ROSEA1* and *RUBY* calli were heavier than CK (*p* = 0.043 and *p* = 0.0036, respectively), and this difference became more pronounced by week 7, when both markers exceeded CK at *p* < 0.001 (Figure 2c). Callus area, however, did not differ significantly among treatments at either timepoint (Figure 2d; week 3, *p* = 0.9; week 7, *p* = 0.83), indicating that the increase in biomass was not accompanied by greater lateral expansion of the callus. Overall, callus induction remained robust across all three constructs, while reporter-specific pigmentation enabled transformed callus to be readily distinguished during proliferation.

### 2.3. Visible Pigmentation Enables Continuous Visual Tracking of Transformed Tissues During Shoot Regeneration

**Figure 3:**
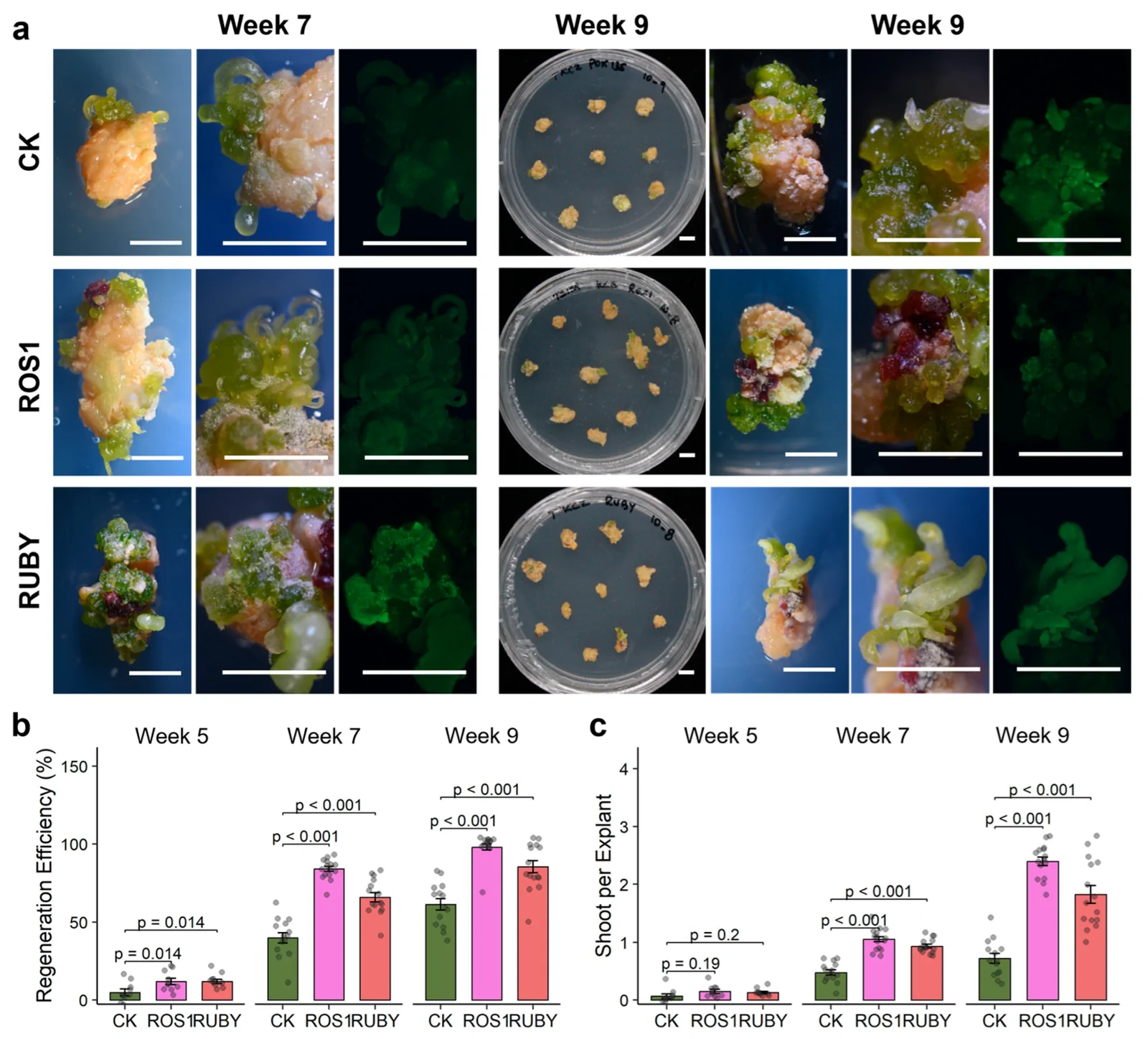
Visible markers *ROSEA1* and *RUBY* indicate plant regeneration status during transformation in citrus. (**a**) Representative phenotypes of citrus explants during *Agrobacterium*-mediated transformation under control (CK), *ROSEA1* (*ROS1*), and *RUBY* treatments at weeks 7 and 9. CK explants showed original primordia without visible pigment accumulation, whereas *ROSEA1* and *RUBY* explants displayed pink-purple anthocyanin and betalain pigmentation, respectively, with colored shoot formation. Representative Petri-dish overviews, close-up views of individual explants, and GFP fluorescence images are shown. (**b**) Regeneration efficiency (%) (**c**) Shoots per explant in CK (green), *ROSEA1* (purple), and *RUBY* (carmine) treatments at weeks 5, 7, and 9. Data are presented as mean ± s.d. (*n* = 8–10 biological replicates, 50–60 explants per treatment, from at least three independent transformation experiments; individual replicate values are overlaid as points). Statistical significance was assessed using two-tailed Student’s *t*-tests (CK versus *ROSEA1*; CK versus *RUBY*). Scale bars, 1 cm (Petri dishes in (**a**)) and 5 mm (close-ups in (**a**)).

Following callus induction, shoot regeneration was compared among CK, *ROSEA1*, and *RUBY* transformed explants on shoot induction medium, and regeneration efficiency and shoot number were scored at weeks 5, 7, and 9. Adventitious shoots developed from callus in all three treatments, exhibiting pink-purple pigmentation in *ROSEA1* tissues and carmine-red in *RUBY* tissues, which also appeared in the developing shoots (Figure 3a). However, CK shoots showed no pigmentation (Figure 3a). GFP fluorescence verified transgene expression in regenerating tissues across all three treatments.

Regeneration efficiency remained relatively low at week 5 but was higher in both reporter treatments than in CK (Figure 3b and Figure S2). These differences became substantially more pronounced at weeks 7 and 9, when both *ROSEA1* and *RUBY* showed markedly higher regeneration efficiencies than CK. Regeneration efficiency in *ROSEA1* and *RUBY* reached approximately 82% and 65% at week 7 and 90% and 82% at week 9, compared with 40% and 62% in CK (*p* < 0.001 for both markers at both time points; Figure 3b). Shoot number followed the same pattern. Shoots per explant did not differ significantly among treatments at week 5 (*p* = 0.19–0.2), but by week 7 both markers exceeded CK (*p* < 0.001), and by week 9 *ROSEA1* and *RUBY* produced approximately 2.4 and 1.8 shoots per explant, respectively, compared with 0.75 in CK (*p* < 0.001; Figure 3c and Figure S4). Overall, both reporter constructs remained compatible with efficient shoot regeneration, while visible pigmentation allowed transformed tissues to be continuously followed during the transition from callus to shoots.

### 2.4. Visible Pigmentation Provides an Alternative to GFP Fluorescence for Identifying Transformed Callus

**Figure 4:**
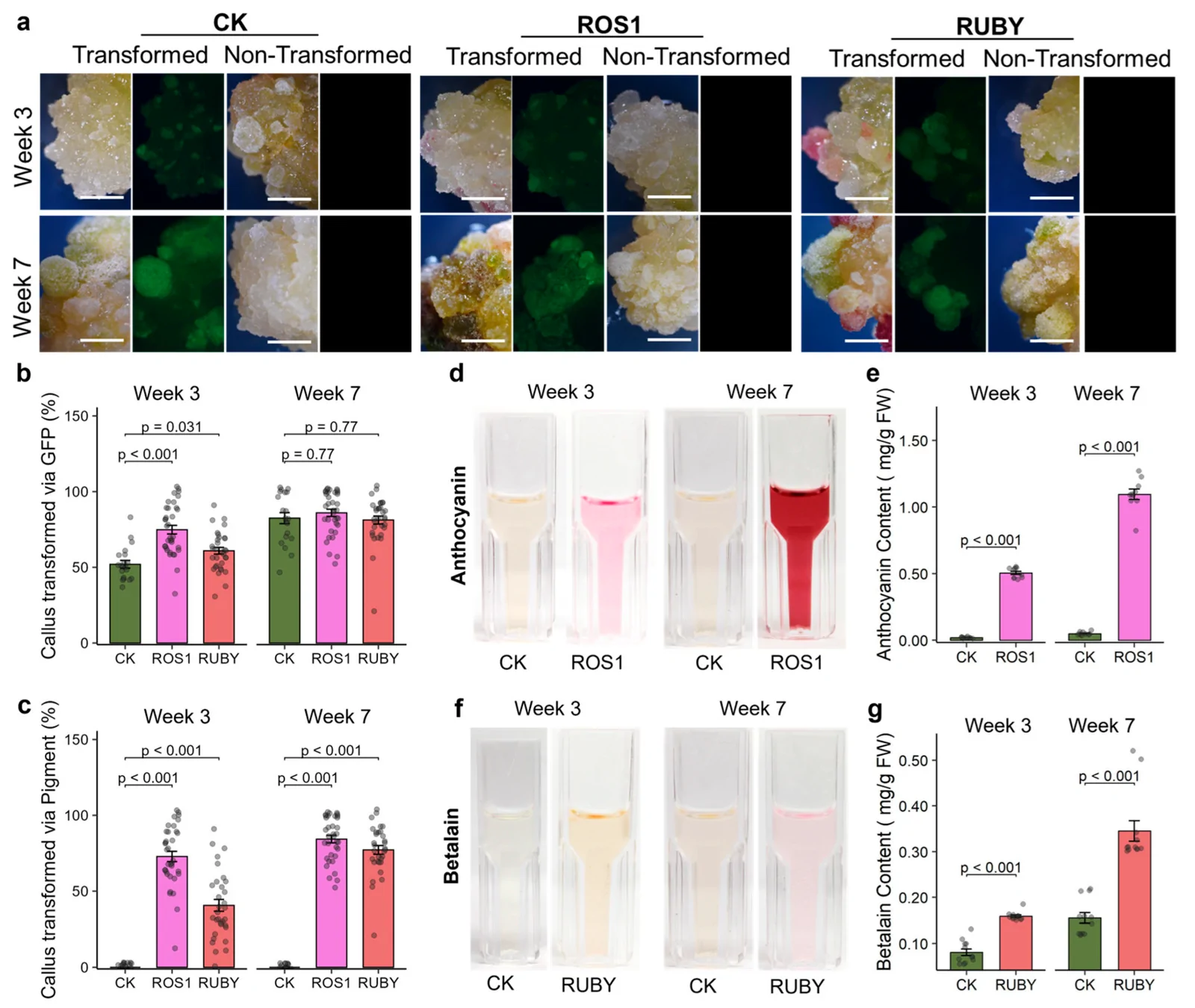
Visible markers *ROSEA1* and *RUBY* indicate callus transformation status in citrus. (**a**) Representative transformed and non-transformed citrus calli during *Agrobacterium*-mediated transformation under control (CK), *ROSEA1* (*ROS1*), and *RUBY* treatments at weeks 3 and 7. Reporter-positive calli showed GFP fluorescence across all three treatments, whereas *ROSEA1*- and *RUBY*-containing calli additionally displayed visible pigmentation. Non-transformed calli lacked GFP fluorescence and visible reporter pigmentation. Scale bar, 5 mm. (**b**,**c**) Callus transformation efficiency, expressed as the percentage of explants cultured that were positive, indicated via GFP fluorescence (**b**) and visible pigmentation (**c**) in CK (green), *ROSEA1* (purple), and *RUBY* (carmine) treatments at weeks 3 and 7. (**d**,**e**) Representative anthocyanin extraction (**d**) and anthocyanin content (mg/g FW) (**e**) in CK and *ROSEA1* treatments at weeks 3 and 7. (**f**,**g**) Representative betalain extraction (**f**) and betalain content (mg/g FW) (**g**) in CK and *RUBY* treatments at weeks 3 and 7. Data are presented as mean ± s.d. (*n* = 8–10 biological replicates, 50–60 explants per treatment, from at least three independent transformation experiments; individual replicate values are overlaid as points). Statistical significance was assessed using two-tailed Student’s *t*-tests (CK versus *ROSEA1*; CK versus *RUBY*). An explant was scored positive if signal was detectable in any sector of the callus. At week 7, GFP-based efficiency reached 83% (CK), 86% (*ROSEA1*) and 82% (*RUBY*), and pigment-based efficiency 85% (*ROSEA1*) and 77% (*RUBY*). Values recorded in CK for betalain content (**g**) reflect background absorbance of the assay and not betalain accumulation, as CK tissues lack the betalain biosynthetic pathway.

After characterizing reporter visibility and regeneration phenotypes at the callus and shoot stages, we next quantitatively compared visible pigmentation with GFP-based detection, beginning with the callus stage. Since all three constructs carry the same eGFP-NPTII cassette, GFP fluorescence served as a common benchmark for evaluating reporter performance. Transformation efficiency was assessed by GFP fluorescence and, for the visible reporter lines, by pigmentation at weeks 3 and 7. Transformed calli showed GFP fluorescence across all treatments, while *ROSEA1* and *RUBY* calli additionally developed pink-purple and carmine-red pigmentation, respectively. In contrast, non-transformed calli showed neither GFP fluorescence nor visible pigmentation (Figure 4a).

GFP-based transformation efficiency varied among treatments at week 3 but increased across all three constructs during continued culture (Figure 4b). By week 7, GFP-based efficiencies had converged to approximately 82–85%, with no significant differences among treatments (*p* = 0.77). Thus, despite differences observed earlier in culture, all three constructs reached comparable GFP-based transformation frequencies by week 7.

Pigment-based scoring detected no positive calli in CK at either timepoint (Figure 4c). In *ROSEA1*, pigment-based detection was already high at week 3 (approximately 74%) and increased to approximately 85% by week 7. *RUBY* pigmentation was detected in a smaller proportion of calli at week 3 (approximately 40%) but increased to approximately 78% by week 7 (*p* < 0.001 versus CK at both timepoints). By week 7, pigment-based and GFP-based detection had converged to comparable levels within each reporter line. In contrast, at week 3, *RUBY* pigmentation lagged behind both its corresponding GFP signal and *ROSEA1* pigmentation, indicating a slower onset of visually detectable betalain accumulation.

To confirm that the visible signals indicated true pigment accumulation, pigments were extracted from callus tissue and quantified. Anthocyanin content in *ROSEA1* was notably higher than in CK at both timepoints and increased markedly between weeks 3 and 7 (Figure 4d,e; *p* < 0.001). Similarly, betalain levels in *RUBY* exceeded those in CK and increased over the same period (Figure 4f,g; *p* < 0.001). This gradual accumulation correlated with the increased detection of pigments from week 3 to 7, establishing a direct link between the visible signals and actual pigment content. CK tissues carry no betalain biosynthetic capacity, and the apparent betalain values recorded in CK represent background absorbance of the assay at 538 and 480 nm, to which endogenous phenolic compounds contribute; the biologically meaningful quantity is therefore the difference between *RUBY* and CK at each timepoint rather than the absolute value. To compare the two detection criteria at the population level, pigment-positive and GFP-positive frequencies were compared within each reporter line. At week 7, *RUBY* pigment-positive and GFP-positive frequencies were 77% and 82%, respectively, while *ROSEA1* frequencies were 85% and 86%, respectively. At week 3, *RUBY* pigmentation remained less frequent than GFP fluorescence (40% versus 61%), whereas *ROSEA1* pigment-positive and GFP-positive frequencies were already similar (73% and 75%).

### 2.5. ROSEA1 and RUBY Show Distinct Visual Detectability During Shoot Regeneration

**Figure 5:**
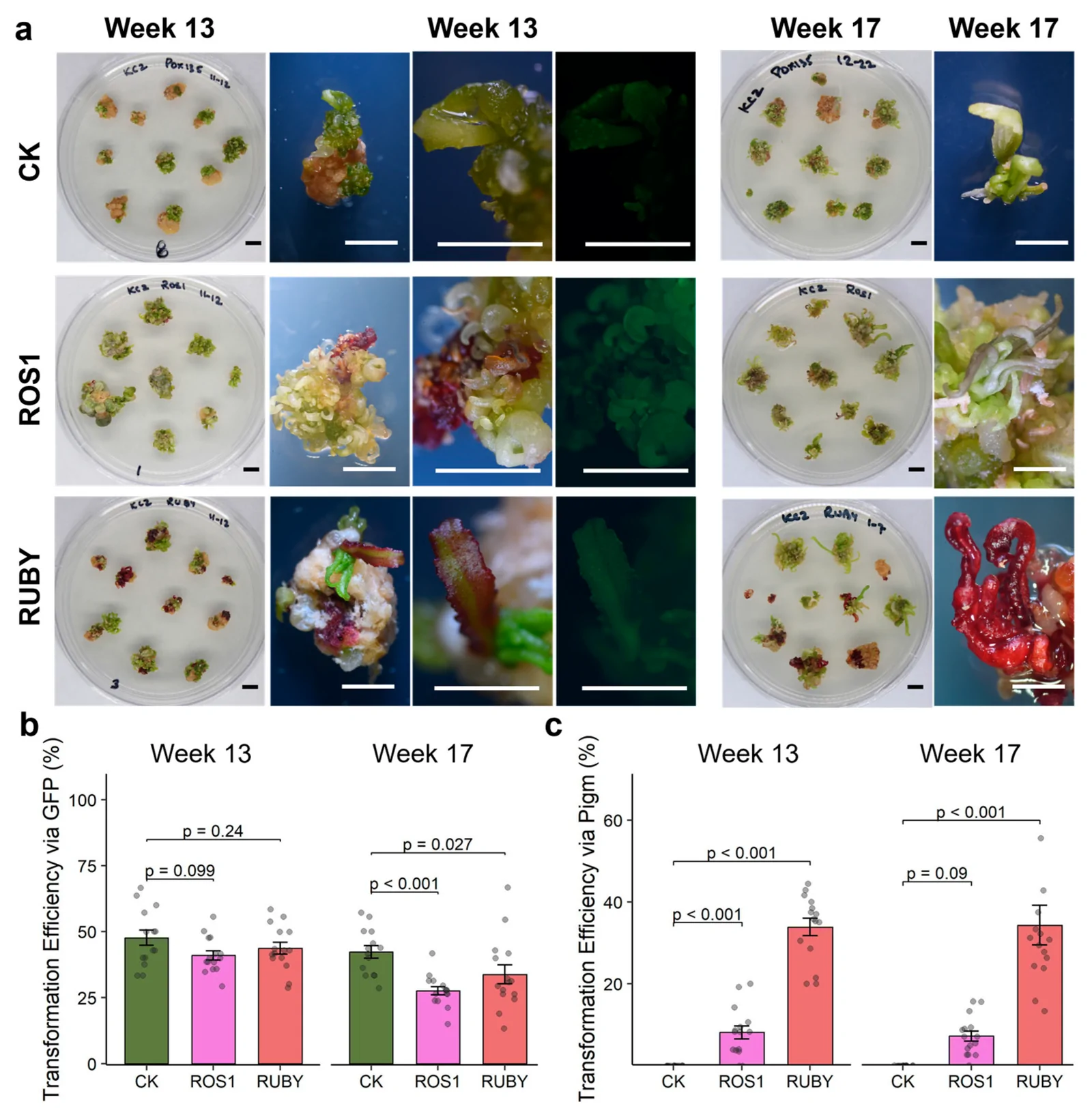
Visible markers *ROSEA1* and *RUBY* indicate shoot transformation status in citrus. (**a**) Representative phenotypes of citrus shoot development during *Agrobacterium*-mediated transformation under control (CK), *ROSEA1* (*ROS1*), and *RUBY* treatments at weeks 13 and 17. CK shoots developed without visible pigment accumulation, whereas *ROSEA1* and *RUBY* shoots displayed pink-purple anthocyanin and betalain pigmentation, respectively. Representative Petri-dish overviews, close-up views of individual shoots, and GFP fluorescence images are shown. (**b**,**c**) Proportion of regenerated shoots that were reporter-positive, indicated via GFP fluorescence (**b**) and visible pigmentation (**c**) in CK (green), *ROSEA1* (purple), and *RUBY* (carmine) treatments at weeks 13 and 17. Data are presented as mean ± s.d. (*n* = 8–10 biological replicates, 50–60 explants per treatment, from at least three independent transformation experiments; individual replicate values are overlaid as points). Statistical significance was assessed using two-tailed Student’s *t*-tests (CK versus *ROSEA1*; CK versus *RUBY*). Panel (**a**) shows representative plates selected to illustrate pigmentation phenotype; it is not intended to convey the frequency of transformed tissue, which is quantified in (**b**,**c**). Arrowheads in (**a**) indicate anthocyanin-containing sectors in *ROSEA1* tissues, shown magnified in the inset. The brownish coloration visible in CK tissues reflects phenolic browning of aging callus and residual explant tissue under prolonged antibiotic selection and is distinct from reporter-derived pigmentation; CK tissues were scored pigment-negative in all replicates. Note that the denominator in (**b**,**c**) is the total number of regenerated shoots, not the number of explants cultured as in Figure 4b,c; these values therefore describe the purity of the recovered shoot population and are not directly comparable with the explant-based efficiencies in Figure 4 or with explant-based values reported elsewhere. Scale bars, 1 cm (Petri dishes in (**a**)) and 5 mm (close-ups in (**a**)).

To determine whether visible pigmentation indicates transformation status in regenerating shoots, where chlorophyll accumulation and tissue differentiation might affect marker visibility compared to callus, transformation was evaluated by GFP fluorescence and visible pigmentation at weeks 13 and 17 (Figure 5a), with week 15 intermediate shoot-stage (Figure S5). *ROSEA1* and *RUBY* shoots showed pink-purple and carmine-red pigmentation, respectively, while CK shoots remained unpigmented. In all three treatments, GFP fluorescence confirmed transformation. Note that shoot-stage values are expressed per regenerated shoot, whereas the callus-stage values in Figure 4 are expressed per explant cultured; shoot-stage values therefore describe the proportion of the recovered shoot population that was transformed and are not directly comparable with the callus-stage efficiencies.

The GFP-positive fraction of regenerated shoots was comparable among the three treatments at week 13 (approximately 42–48%; Figure 5b; *p* = 0.099 and *p* = 0.24 for *ROSEA1* and *RUBY* versus CK) but declined by week 17, when both marker lines were significantly lower than CK (*ROSEA1*, *p* < 0.001; *RUBY*, *p* = 0.027). This decline in GFP-based efficiency occurred despite all constructs carrying the same eGFP-NPTII cassette. Expressed as a proportion of regenerated shoots, 52 to 59% of shoots recovered at week 13 under continuous kanamycin selection lacked detectable GFP and were therefore presumptively non-transformed; because GFP-negative status may also arise from transgene silencing, this represents an upper bound on escape frequency.

Pigment-based scoring produced no positive shoots in CK at either timepoint, confirming marker specificity, but the two pigment markers diverged markedly (Figure 5c). *RUBY*-based detection reached approximately 34% at both timepoints (*p* < 0.001 versus CK), comparable to the GFP-based efficiency in the same line by week 17. *ROSEA1*-based detection, by contrast, remained low throughout, at approximately 8% at week 13 and 7% at week 17, and was significantly above CK only at week 13 (*p* < 0.001), with the difference no longer significant by week 17 (*p* = 0.09). Anthocyanin pigmentation therefore identified only a small proportion of transformed shoots at this stage, well below the corresponding GFP-based efficiency. These results indicate that, in contrast to the callus stage, at which both markers reported transformation efficiently, only *RUBY* provided visual detection of transformed shoots comparable to GFP, whereas *ROSEA1*-based detection was substantially reduced during shoot regeneration. At week 17, the *RUBY* pigment-positive fraction (approximately 34%) was similar to the GFP-positive fraction in the same treatment (approximately 33.5%), whereas the *ROSEA1* pigment-positive fraction remained substantially lower than the corresponding GFP-positive fraction at both week 13 and week 17. *RUBY* pigmentation was readily visible across reporter-positive calli and reporter-positive shoots at week 17, where the two detection frequencies were similar; we do not extend this observation to *ROSEA1* at the shoot stage, where pigmentation was restricted to a minority of GFP-positive shoots, nor to earlier *RUBY* timepoints.

### 2.6. Recovery and Molecular Confirmation of Stable Transgenic Plantlets

**Figure 6:**
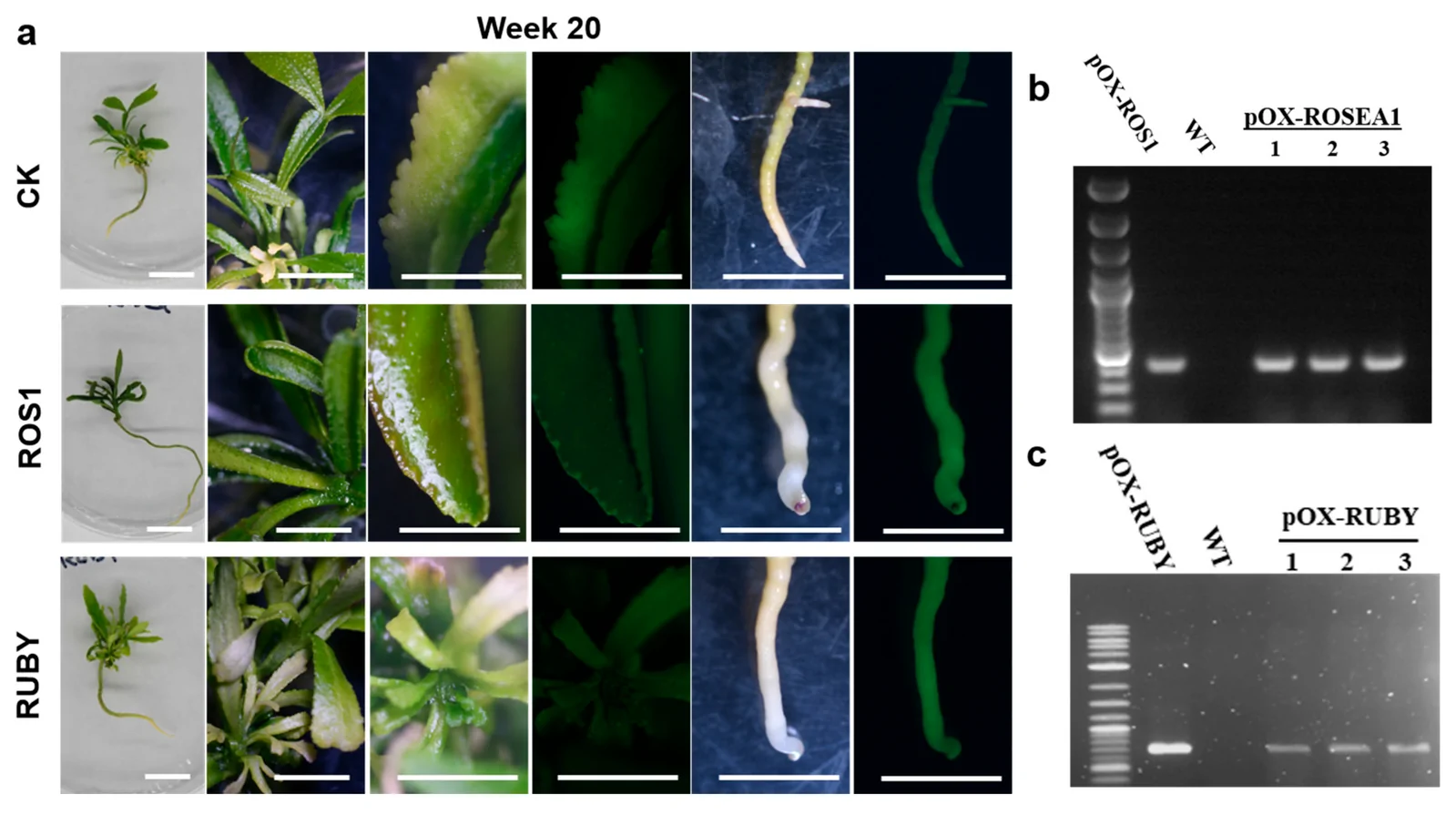
Transgenic citrus plantlets stably expressing *ROSEA1* and *RUBY* visible markers at week 20. (**a**) Representative fully developed transgenic citrus plantlets under control (CK), *ROSEA1* (*ROS1*) and *RUBY* treatments at week 20; overview images, close-up views and GFP fluorescence detection in leaves and root tips are shown. Reporter pigmentation in recovered plantlets was localised rather than distributed throughout the plant: in *RUBY* plantlets it was present at the apical meristem, the bases of expanding leaves and the petioles of the youngest leaves, and in *ROSEA1* plantlets at occasional leaf margins and root tips. Fully expanded leaves appeared green in all treatments, and no reporter pigmentation was present in CK. (**b**,**c**) PCR genotyping of genomic DNA from independent regenerated lines using *ROSEA1*-specific (**b**) and *RUBY*-specific (**c**) gene-internal primers. Lanes: M, GeneRuler 1 kb Plus DNA Ladder (Thermo Fisher); P, plasmid positive control (pOX-*ROSEA1* or pOX-*RUBY*); WT, non-transformed Carrizo citrange; 1–3, three independent transgenic lines. Expected amplicon sizes are 450 bp (*ROSEA1*) and 700 bp (*RUBY*). Scale bars, 1 cm (whole plantlets in (**a**)) and 5 mm (close-ups in (**a**)).

Rooted plantlets were recovered from all three treatments and developed into fully formed plantlets by week 20 (Figure 6a and Figure S6). GFP fluorescence was detected in the leaves and root tips of plantlets from all three treatments, consistent with stable expression of the shared eGFP-NPTII cassette in differentiated tissues (Figure 6a). Reporter pigmentation persisted in recovered plantlets but was localised rather than distributed throughout the plant. In *RUBY* plantlets, betalain was visible at the apical meristem, the bases of expanding leaves, and the petioles of the youngest leaves; in *ROSEA1* plantlets, anthocyanin was visible at occasional leaf margins and at root tips (Figure 6a). Fully expanded leaves appeared green in all three treatments. No reporter pigmentation was observed in CK at any site. The presence of the transgenes in regenerated plantlets was confirmed by PCR genotyping of genomic DNA from independent regenerated lines (Figure 6b,c). Amplification with *ROSEA1*- and *RUBY*-specific primers yielded products of the expected size in three independent pOX-*ROSEA1* lines and three independent pOX-*RUBY* lines, respectively, corresponding to the amplicons obtained from the positive plasmid controls, whereas no product was amplified from wild-type genomic DNA in either assay (Figure 6b,c). Taken together with the GFP fluorescence detected in leaves and root tips, these results confirm the recovery of stably transformed citrus plantlets carrying the *ROSEA1* or *RUBY* visible marker.

## 3. Discussion

Citrus is widely regarded as recalcitrant to *Agrobacterium*-mediated transformation, with success rates ranging from 0 to about 45% and strongly dependent on factors such as species, genotype, explant type, and selection strategy [11,13,20,29]. In citrus the principal constraint is often not gene delivery itself but the reliable identification of transformed tissue, because during prolonged regeneration antibiotic selection permits non-transformed escapes and chimeric shoots to persist [10,18]. Scorable reporter genes that permit visual identification of transformed tissue offer a direct means of addressing this bottleneck [21,30]. Here, we established an indirect organogenesis-based transformation workflow for Carrizo citrange and evaluated *ROSEA1* and *RUBY* as visible reporters for tracking transformed tissues throughout the regeneration process using a shared GFP–NPTII cassette as a common fluorescence benchmark.

The workflow supported callus induction within one week of transfer to selective medium, shoot regeneration from week 5, and recovery of rooted, PCR-confirmed transgenic plantlets by week 20. Two features distinguish it from the direct-organogenesis protocols that predominate in citrus. First, shoots arise from a proliferative callus phase rather than directly from wounded explant tissue. In direct organogenesis adventitious shoots develop from multiple neighbouring competent cells, which increases the likelihood of recovering chimeric regenerants [11,18,31,32]; an intervening callus phase allows transformed cells to expand clonally before organogenic commitment, providing a biological basis for recovering more uniformly transformed regenerants [17,33,34]; consistent with this, embryogenic cell cultures have been used to recover genome-edited citrus lines without chimerism [35]. Second, regenerated shoots rooted readily on IBA-containing medium without shoot-tip micrografting, which many citrus protocols require because adventitious rooting is poor [12,22], simplifying the workflow and shortening recovery time.

Both reporters were compatible with the full regeneration sequence. Callus induction efficiency and callus area were equivalent across treatments, callus fresh weight was higher in the reporter lines, and regeneration efficiency and shoot number were higher in the reporter treatments than in the control from week 7 onward. We draw no mechanistic conclusion from these differences. They were secondary observations from a design not constructed to test them, and they cannot be separated from preferential recovery of transformed, regeneration-competent tissue under continuous selection, since non-transformed cells may persist within or adjacent to transformed tissue under kanamycin [18,32]. The conclusion we draw is therefore one of compatibility rather than of benefit or detriment: neither *ROSEA1* nor *RUBY* impaired callus establishment, callus proliferation or shoot formation. Reporter expression can affect regeneration in some transformation systems [36,37], emphasizing the importance of confirming reporter compatibility in each species.

The two reporters diverged after shoot induction. *RUBY* maintained consistent pigmentation throughout regeneration, whereas *ROSEA1*-associated pigmentation declined despite continued GFP fluorescence and PCR confirmation of the transgene. The mechanism underlying this decline was not determined here. Loss of visible pigmentation alongside retained GFP signal is compatible with developmental regulation of anthocyanin accumulation, with transcriptional silencing of the *ROSEA1* cassette, or with both, and pigment and fluorescence measurements cannot distinguish among them; because eGFP-NPTII is driven by the CsVMV promoter whereas the reporter cassette is under a 2×CaMV35S promoter with an Omega enhancer (Section 4.4), persistence of GFP does not exclude promoter-specific silencing of the reporter. RT-qPCR quantification of transgene transcript across the regeneration time course would discriminate these possibilities and is the necessary next experiment. The contrast is nonetheless consistent with the underlying biology: *ROSEA1* acts through the endogenous MYB-bHLH-WD40 complex, whose activity depends on tissue identity, developmental stage and light [25,38], and anthocyanin accumulation in citrus has been reported to be stronger in juvenile than in mature tissue [39,40,41]; this developmental pattern is specific to the anthocyanin pathway and is not expected to apply to betalain accumulation in the same way, since *RUBY* encodes the complete betalain pathway as a polycistronic cassette and synthesises pigment independently of endogenous regulators [21]. In recovered plantlets, pigmentation was localised to meristematic and expanding tissue rather than distributed throughout the plant, and expanded leaves appeared green in all treatments; because pigment was quantified in callus tissue only, we cannot distinguish reduced accumulation from optical masking by chlorophyll at this stage.

Escapes were not eliminated by antibiotic selection. Under continuous kanamycin, 52 to 59% of regenerated shoots at week 13 lacked detectable GFP and were therefore presumptively non-transformed, consistent with reports for citrus [12,18]; because GFP-negative status may also arise from silencing, this is an upper bound. What a visible reporter contributes is the ability to act on reporter-positive tissue at each subculture without instrumentation. For *RUBY*, pigment-positive and GFP-positive frequencies were closely similar by week 7 and remained similar at week 17, whereas *ROSEA1* pigmentation was substantially less frequent than GFP fluorescence during shoot regeneration. Visibly mosaic tissue was infrequent in this system; we report this as a qualitative observation from routine assessment at each subculture rather than as a quantified result, since sectorial pigmentation was not scored systematically. Three limits should be stated plainly. Chimerism was not quantified; surface scoring of pigment could not in any case exclude unpigmented cells internal to a pigmented unit, so any such measure would be a lower bound on true mosaicism and an upper bound on uniformity, and definitive assessment of clonal origin would require histological sectioning or segregation analysis in progeny; and because the study included no direct-organogenesis or selection-only comparison arm, whether this workflow reduces the eventual recovery of escapes and chimeric plants relative to antibiotic selection alone remains to be demonstrated.

For practical application in Carrizo citrange we recommend the following scheme. Use *RUBY* as the visible reporter when monitoring is required beyond the callus stage. Score calli under ambient light at every two-week subculture from week 3, but treat the absence of pigmentation before week 5 as provisional and retain unpigmented calli at that stage, because *RUBY* pigmentation lags GFP detection early in culture. From week 7 onward, visibly pigmented sectors can be preferentially excised and subcultured separately. Where maximizing recovery of independent transformation events is important, unpigmented tissue can be retained temporarily or checked by GFP before being discarded. During shoot regeneration, *RUBY*-positive shoots can be prioritized for elongation and rooting, whereas visibly sectorial shoots can be re-excised or subjected to additional selection. All recovered plantlets should be confirmed molecularly before use. This scheme is intended to enrich reporter-positive tissue during prolonged culture rather than to increase absolute transformation frequency or eliminate chimerism, neither of which was tested here. These recommendations apply to any construct delivered on a T-DNA carrying the reporter, including overexpression, silencing and editing constructs, because the reporter reports on delivery and expression of the T-DNA rather than on the function of the gene of interest, and visible pigmentation is in all cases a means of enriching reporter-positive tissue during culture rather than a substitute for molecular confirmation. Finally, this system was developed and validated in Carrizo citrange, which is among the most readily transformed and most regenerable citrus genotypes. Regeneration competence, endogenous anthocyanin regulatory capacity and reporter visibility are all strongly genotype-dependent in citrus [11,12]; extension to commercially important scion cultivars cannot be assumed and represents a genuine challenge for future work, and *ROSEA1* in particular may behave differently in genotypes with a different endogenous MYB-bHLH-WD40 background.

## 4. Materials and Methods

### 4.1. Plant Material and Seed Sterilization

Carrizo citrange (*Citrus sinensis* × *Poncirus trifoliata*) seeds were obtained from Lyn Citrus Seeds (Arvin, CA, USA). Seeds were surface sterilized under aseptic conditions in a laminar airflow cabinet. Sterilization involved immersion in 70% (*v*/*v*) ethanol for 1 min, followed by 20% (*v*/*v*) commercial bleach solution (approximately 1% sodium hypochlorite) containing 2–3 drops of Tween-20 for 15 min with gentle agitation. Seeds were subsequently rinsed seven times with sterile distilled water to remove residual bleach and surfactant and were blotted dry on sterile filter paper prior to inoculation.

### 4.2. In Vitro Germination

Sterilized seeds were cultured on solid Murashige and Skoog (MS) basal medium [42] supplemented with 3% (*w*/*v*) sucrose and solidified with 0.7% (*w*/*v*) agar. The pH was adjusted to 5.8 prior to autoclaving at 121 °C for 15 min. Cultures were incubated in complete darkness at 25 °C for 6 weeks. Germinated seedlings were subsequently maintained under dark conditions for an additional 7 days to promote etiolation and epicotyl elongation. Etiolated seedlings served as the source of explants for transformation experiments.

### 4.3. Explant Preparation

The epicotyl region (defined as the segment between the cotyledons and the first pair of true leaves) was excised from 7-week-old seedlings and sectioned into 5–7 mm segments using a sterile scalpel and forceps. To enhance *Agrobacterium* accessibility, both ends of each epicotyl segment were lightly wounded. Epicotyl segments were pre-cultured on callus induction medium (CIM0) without antibiotics for 5 days in complete darkness at 25 °C.

### 4.4. Vector Construction

All constructs were based on the pOX135 binary vector [43], which carries a fused eGFP-NPTII gene under the control of the Cassava vein mosaic virus (CsVMV) promoter and terminated by the 35S terminator (35S-T), enabling both fluorescent detection and kanamycin selection, together with a spectinomycin resistance gene for bacterial selection. Coding sequences were introduced at the BsaI cloning sites under a 2×CaMV35S promoter containing an Omega enhancer (pGWB402, AB294426.1) and terminated by the heat-shock protein terminator (Hsp-T).

Two visible reporters were used. *ROSEA1* encodes an R2R3-MYB transcription factor from *Antirrhinum majus* that activates anthocyanin biosynthesis through the endogenous MYB-bHLH-WD40 (MBW) complex [25]; the coding sequence corresponds to GenBank AKB94073.1 and was commercially synthesized (Gene Universal Inc., Newark, DE, USA). *RUBY* is a synthetic cassette encoding three betalain biosynthetic enzymes from *Beta vulgaris*, CYP76AD1, DODA1 and a cyclo-DOPA 5-O-glucosyltransferase linked by P2A self-cleaving peptides, which together convert tyrosine to betalain; the cassette was amplified from plasmid 35S:*RUBY* (Addgene #160908; He et al. [21]). The resulting constructs were designated pOX135-*ROSEA1* and pOX135-*RUBY*, and the unmodified pOX135 vector served as the control (CK).

Each construct was verified by PCR across the expression cassette (Figure 1d) and confirmed by Sanger sequencing. Verification used a single cassette-flanking primer pair common to all constructs, giving products of 1.1, 1.7 and 3.2 kb for CK, pOX135-*ROSEA1* and pOX135-*RUBY* respectively, and Sanger sequencing covered the full expression cassette including both insertion junctions. Verified plasmids were introduced into *Agrobacterium tumefaciens* strain EHA105 by the freeze–thaw method, and transformed colonies were selected on LB medium supplemented with spectinomycin (100 mg L^−1^) and rifampicin (50 mg L^−1^), verified by colony PCR, and maintained as glycerol stocks at −80 °C.

### 4.5. *Agrobacterium*-Mediated Transformation

*Agrobacterium tumefaciens* strain EHA105 harboring the binary vectors was cultured in liquid yeast extract beef (YEB) medium supplemented with 100 mg L^−1^ spectinomycin and 25 mg L^−1^ rifampicin at 28 °C with shaking at 200 rpm overnight. A secondary culture was initiated by transferring 1 mL of the overnight culture into 10 mL of fresh LB broth containing the same antibiotics and grown until the OD600 reached 0.3–0.5 (mid-log phase). Bacterial cells were pelleted by centrifugation at 4000× *g* for 10 min and resuspended in infection buffer (½-strength MS salts supplemented with 10 mM MES, pH 5.8, 20 g L^−1^ sucrose, and 100 µM acetosyringone) to an OD_600_ of 0.2 for plant infection. Pre-cultured epicotyl explants were immersed in the *Agrobacterium* suspension for 5 min with gentle agitation to ensure uniform contact. Following infection, explants were blotted dry on sterile filter paper and placed on co-cultivation medium (CIM supplemented with 100 µM acetosyringone and without antibiotic supplementation). Co-cultivation was carried out for 48 h in the dark at 25 °C.

### 4.6. Callus Induction and Selection

Following co-cultivation, explants were transferred to callus induction medium (CIM) to select for transformed tissue. CIM had the same composition as the pre-culture medium (CIM0)—full-strength MS salts and vitamins, 3% (*w*/*v*) sucrose, 1 mg L^−1^ 6-benzylaminopurine (BAP), 0.2 mg L^−1^ 1-naphthaleneacetic acid (NAA), and 0.5 mg L^−1^ 2,4-dichlorophenoxyacetic acid (2,4-D), pH 5.8, with the addition of 75 mg L^−1^ kanamycin to select transformed cells and 200 mg L^−1^ Timentin, to suppress residual *Agrobacterium*. Explants were maintained in darkness at 25 °C and subcultured onto fresh CIM every 2 weeks. Callus induction efficiency, callus fresh weight, and callus area were recorded at weeks 3 and 7 (Figure 2). Callus induction efficiency was calculated as

Callus induction efficiency (%) = Number of explants forming callus/Total number of explants cultured × 100

Callus fresh weight was determined by excising callus from the explant and weighing immediately on an analytical balance and is reported as g per explant.

Callus area was measured from calibrated overhead images in ImageJ 1.46r (NIH, Bethesda, MD, USA) against an in-frame scale reference and is reported as cm^2^ per explant.

### 4.7. Shoot Regeneration and Elongation

Actively growing callus was excised and transferred to shoot induction medium (SIM) containing MS salts and vitamins, 3% (*w*/*v*) sucrose, and 0.5 mg L^−1^ Thidiazuron (TDZ), 0.8% (*w*/*v*) agar, 2 mg L^−1^ silver nitrate, and 5% (*v*/*v*) coconut water, with the pH adjusted to 5.8, along with Kanamycin (75 mg L^−1^) and Timentin (200 mg L^−1^). Developing shoots were sub-cultured onto fresh SIM every 2 weeks. Regenerated shoots (≥1 cm) were transferred to shoot elongation medium (SEM: MS salts and vitamins, 3% (*w*/*v*) sucrose, 0.5 mg L^−1^ BAP, 0.25 mg L^−1^ gibberellic acid (GA_3_), pH 5.8, with the same antibiotics) to promote elongation. Regeneration efficiency and shoots per explant were calculated as

Regeneration efficiency (%) = Number of explants producing shoots/Total number of explants cultured × 100

Shoots per explant = Total number of shoots/Total number of explants cultured

### 4.8. Rooting and Acclimatization

Elongated shoots were transferred to rooting medium (½-strength MS salts, 2% (*w*/*v*) sucrose, 0.5 mg L^−1^ indole-3-butyric acid (IBA), pH 5.8) under a 16 h photoperiod at 25 °C. The medium was supplemented with Kanamycin (75 mg L^−1^) and Timentin (200 mg L^−1^). Successfully rooted plantlets were gradually acclimatized by transferring them to sterile substrate under high humidity and subsequently maintained under controlled greenhouse conditions. Rooting efficiency was calculated as

Rooting efficiency (%) = Number of shoots forming roots/Total number of shoots transferred to rooting medium × 100

### 4.9. Assessment of Transformation Efficiency

Transformation efficiency was assessed using both GFP fluorescence and visible pigmentation. GFP fluorescence was detected using a fluorescence stereomicroscope (Leica M205 FA, Leica Microsystems, MA, USA) fitted with a GFP filter set; pigmentation was scored macroscopically under ambient light. Scoring was performed at the callus stage at weeks 3 and 7, and at the shoot stage at weeks 13 and 17. Scoring was binary at the level of the individual explant (callus stage) or the individual regenerated shoot (shoot stage): a callus was recorded as positive if GFP fluorescence, or reporter-specific pigmentation, was detectable in any sector of the tissue, so that partial or sectorial signal was scored as positive; a shoot was recorded as positive if the signal was detectable in the shoot itself rather than only in the callus from which it arose. The values reported therefore represent the frequency with which transformation events were recovered, and not the proportion of transformed cells within a given tissue. A callus showing only a pigmented sector was advanced by excising that sector rather than being carried forward whole. Scoring was performed at the bench at each assessment, and close-up images were captured for representative units rather than for every unit scored, so units cannot be reclassified retrospectively into uniform and sectorial categories; prospective sector-level scoring is included in the recommended scheme set out in the Discussion. Regenerated shoots that developed under continuous kanamycin selection but lacked detectable GFP fluorescence were recorded as presumptive escapes. Images were acquired under standard ambient laboratory illumination; brightness and contrast adjustments, where applied, were applied uniformly across the entire image and identically to all treatments within a panel. Two denominators were used, and they are not interchangeable. At the callus stage, efficiency was expressed per explant cultured, which corresponds to the convention used in the citrus transformation literature and is unaffected by the recovery of non-transformed escape tissue. At the shoot stage, efficiency was expressed per regenerated shoot; this quantity measures the proportion of the recovered shoot population that was transformed rather than productivity per explant, and it decreases as non-transformed escape shoots are recovered. Values calculated on these two bases should not be compared directly with one another, nor should the shoot-stage values be compared with explant-based transformation efficiencies reported elsewhere. Efficiency at each stage was calculated as

Transformation efficiency callus (%) = Number of explants positive for GFP (or pigment)/Total number of explants cultured × 100

Shoot reporter-positive fraction (%) = Number of shoots positive for GFP (or pigment)/Total number of regenerated shoots × 100

At each timepoint, 8–10 culture plates and 50–60 explants per treatment were scored, across at least three independent transformation experiments.

### 4.10. Anthocyanin and Betalain Quantification

Anthocyanin content was determined spectrophotometrically following established protocols [44]. Approximately 100 mg of callus tissue was homogenized in 1 mL of acidified methanol (1% HCl) and incubated overnight at 4 °C in the dark. Samples were centrifuged at 12,000× *g* for 10 min, and the absorbance of the supernatant was measured at 530 nm and 657 nm using a UV-visible spectrophotometer (NanoDrop 2000, ThermoFisher, Wilmington, NC, USA).

Anthocyanin content was calculated using the formula: A_530_ − 0.25 × A_657_, and expressed as mg g^−1^ fresh weight (FW).

Total betalain content was quantified as described by [45]. Callus tissue (100 mg) was homogenized in distilled water and centrifuged at 12,000× *g* for 10 min. Absorbance of the supernatant was measured at 538 nm (betacyanins) and 480 nm (betaxanthins). Total betalain content was calculated and expressed as mg g^−1^ FW. Because this spectrophotometric method reports total absorbance at the betacyanin and betaxanthin wavelengths, non-transformed tissues yield non-zero values arising from endogenous compounds absorbing in the same range; values recorded from CK tissues therefore represent the assay background rather than betalain content. Betalain content was calculated using the formula:

Betalain content (mg/g FW) = (A × DF × MW × V)/(ε × L × W),

where A = absorbance, DF = dilution factor, MW = molecular weight (550 g/mol for betacyanins; 308 g/mol for betaxanthins), V = extract volume (mL), ε = molar extinction coefficient (60,000 L/mol·cm for betacyanins; 48,000 L/mol·cm for betaxanthins), L = path length (1 cm), and W = fresh weight (g) [46].

### 4.11. Molecular Confirmation of Transformants

Putative transformation events growing on kanamycin selection medium were further validated by PCR. Genomic DNA was isolated from 100 mg of leaf tissue using a modified cetyltrimethylammonium bromide (CTAB) method [47]. Genotyping used gene-internal primers targeting *ROSEA1* and *RUBY* (Table S1), distinct from the cassette-flanking primer pair used for plasmid verification in Figure 1d, and yielding single products of 450 bp and 700 bp respectively. Amplifications were performed with Taq DNA polymerase (New England Biolabs, Ipswich, MA, USA) on a Bio-Rad thermocycler in 25 µL reactions containing 100 ng genomic DNA, 1X Taq buffer, 0.2 mM of each dNTP and 0.2 µM of each primer, under the following conditions: initial denaturation at 95 °C for 10 min; 30 cycles of 95 °C for 30 s, 52 °C for 30 s and 72 °C for 45 s; and a final extension at 72 °C for 5 min. Wild-type (non-transformed) Carrizo citrange served as the negative control and plasmid DNA as the positive control. Products were separated on 1.0% (*w*/*v*) agarose gels in 1× TAE alongside GeneRuler 1 kb Plus DNA Ladder (New England Biolabs, Ipswich, MA, USA) stained with SYBR Safe DNA Gel Stain and imaged on a Gel-Doc imager (Thermo Fisher Scientific, Waltham, MA, USA).

### 4.12. Experimental Design and Statistical Analysis

Each independent transformation experiment included three treatments—the control vector (CK), pOX135-*ROSEA1* (*ROS1*), and pOX135-*RUBY*. and the experiment was repeated at least three times. Across these independent experiments, a biological replicate was defined as one culture plate; and each treatment comprised 8–10 replicate plates and a total of 50–60 explants. Unless otherwise indicated, experimental week numbers were counted from the transfer of co-cultivated explants to selective CIM, which was designated as week 0. Callus induction efficiency, callus fresh weight, and callus area were recorded at weeks 3 and 7; regeneration efficiency and shoots per explant at weeks 5, 7, and 9; and callus and shoot transformation efficiency, scored by GFP fluorescence and by visible pigmentation, at weeks 3 and 7 and weeks 13 and 17, respectively. Anthocyanin content was quantified in CK and *ROSEA1* tissues, and betalain content in CK and *RUBY* tissues, at weeks 3 and 7.

Data are presented as mean ± standard deviation (s.d.). Statistical significance was assessed using two-tailed Student’s *t*-tests (CK versus *ROSEA1*; CK versus *RUBY*). A *p*-value < 0.05 was considered statistically significant. All analyses were performed in R (version 4.3.2), and figures were generated using ggplot2. Efficiency values expressed per explant (callus stage) and per regenerated shoot (shoot stage) were computed separately for each culture plate and then averaged across plates; summary values are reported as the mean of plate-level values.

## 5. Conclusions

In this study, we established an *Agrobacterium*-mediated indirect organogenesis-based transformation system for Carrizo citrange that supported callus induction, shoot regeneration, rooting without micrografting, and recovery of PCR-confirmed transgenic plantlets within approximately 20 weeks. Integration of *ROSEA1* and *RUBY* visible reporters enabled transformed tissues to be monitored non-destructively from callus proliferation through shoot regeneration, the stages at which selection decisions are made, with GFP providing an independent fluorescence-based reference. Both reporters performed effectively during the callus stage, while *RUBY* maintained more consistent visual detectability during shoot regeneration than *ROSEA1*. Importantly, neither reporter prevented efficient regeneration nor recovery of transgenic plants. Overall, this work provides a practical indirect organogenesis-based platform for citrus transformation and demonstrates the value of visible pigmentation as an equipment-free complement to antibiotic selection and fluorescence-based screening during prolonged regeneration. The system provides a useful foundation for future citrus functional genomics and genome-editing applications. On the basis of these results, *RUBY* is recommended as the visible reporter of choice for citrus transformation extending beyond the callus stage. The workflow was established in Carrizo citrange, among the most readily transformed citrus genotypes, and its extension to other varieties remains to be demonstrated.

## Data Availability

All data supporting the findings of this study are available from the corresponding author upon reasonable request.

## Supplementary Materials

The following supporting information can be downloaded at https://www.mdpi.com/article/10.3390/plants15203141/s1: Supplementary Figure S1: Representative citrus explants during *Agrobacterium*-mediated transformation at week 3. Additional biological replicates of citrus explants under control (CK), *ROSEA1* (*ROS1*), and *RUBY* treatments at week 3. Five representative plates per treatment are shown. Scale bars, 1 cm. Supplementary Figure S2: Representative citrus explants during *Agrobacterium*-mediated transformation at week 5. Additional biological replicates of citrus explants under control (CK), *ROSEA1* (*ROS1*), and *RUBY* treatments at week 5. Five representative plates per treatment are shown. Scale bars, 1 cm. Supplementary Figure S3: Representative citrus explants during *Agrobacterium*-mediated transformation at week 7. Additional biological replicates of citrus explants under control (CK), *ROSEA1* (*ROS1*), and *RUBY* treatments at week 7. Five representative plates per treatment are shown. Scale bars, 1 cm. Supplementary Figure S4: Representative citrus explants during *Agrobacterium*-mediated transformation at week 9. Additional biological replicates of citrus explants under control (CK), *ROSEA1* (*ROS1*), and *RUBY* treatments at week 9. Five representative plates per treatment are shown. Scale bars, 1 cm. Supplementary Figure S5: Representative citrus explants during *Agrobacterium*-mediated transformation at week 15. Additional biological replicates of citrus explants under control (CK), *ROSEA1* (*ROS1*), and *RUBY* treatments at week 15. Four representative plates per treatment are shown. Scale bars, 1 cm. Supplementary Figure S6: Representative fully developed wild-type citrus plantlets at week 20. Whole-plant overview (left), close-up leaf images (middle), and GFP fluorescence (right) imaging of leaves are shown. Scale bars, 1 cm. Supplementary Table S1: The list of primers used in this study.

## Abbreviations

The following abbreviations are used in this manuscript:

BAP	6-Benzylaminopurine
CCM	Co-cultivation medium
CIM	Callus induction medium
CLas	*Candidatus Liberibacter asiaticus*
eGFP	Enhanced green fluorescent protein
HLB	Huanglongbing
IBA	Indole-3-butyric acid
NAA	1-Naphthaleneacetic acid
NPTII	Neomycin phosphotransferase II
RIM	Rooting medium
SEM	Shoot elongation medium
SIM	Shoot induction medium
TDZ	Thidiazuron
