# Supplementary material for: An Indirect Organogenesis-Based Citrus Transformation System Monitored Using Visible Reporter Markers
Source: Plants (Basel). 2026 Oct 14;15(20):3141. doi: 10.3390/plants15203141 (PMC13708800; doi:10.3390/plants15203141)
Supplement: Supplementary file 1 [file plants-15-03141-s001.zip › plants-4581998-supplementary.pdf]

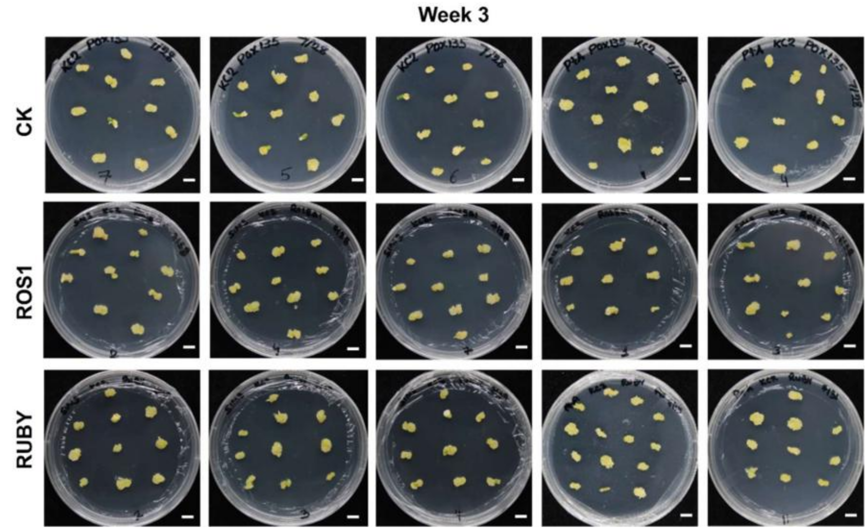

Supplementary Figure S1. Representative citrus explants during Agrobacterium-mediated transformation at week 3. Additional biological replicates of citrus explants under control (CK), ROSEA1 (ROS1), and RUBY treatments at week 3. Five representative plates per treatment are shown. Scale bars, 1 cm.

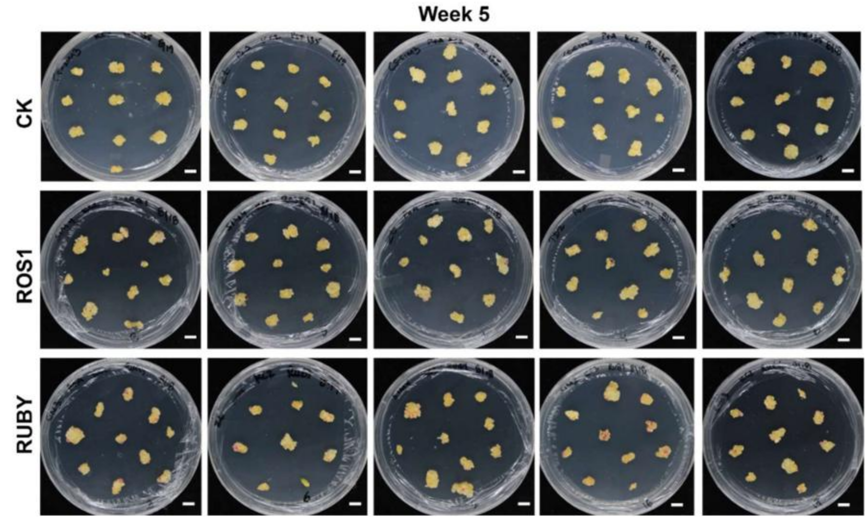

Supplementary Figure S2. Representative citrus explants during Agrobacterium-mediated transformation at week 5. Additional biological replicates of citrus explants under control (CK), ROSEA1 (ROS1), and RUBY treatments at week 5. Five representative plates per treatment are shown. Scale bars, 1 cm.

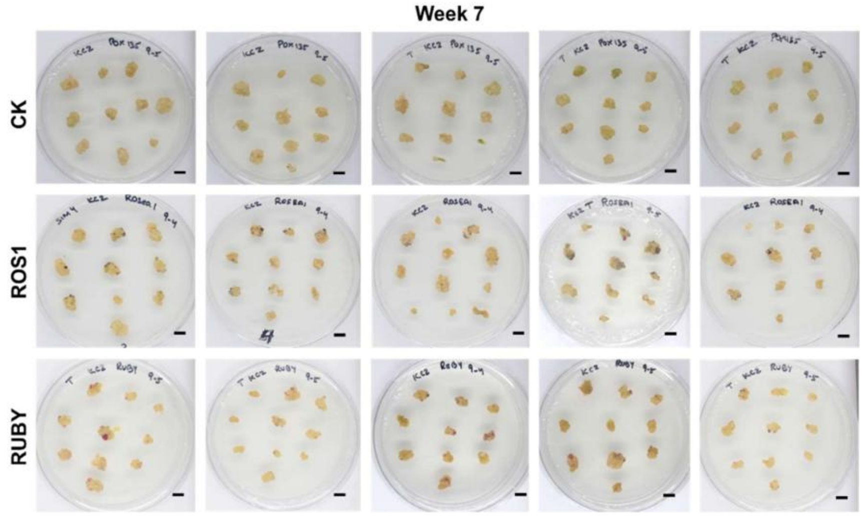

Supplementary Figure S3. Representative citrus explants during Agrobacterium-mediated transformation at week 7. Additional biological replicates of citrus explants under control (CK), ROSEA1 (ROS1), and RUBY treatments at week 7. Five representative plates per treatment are shown. Scale bars, 1 cm.

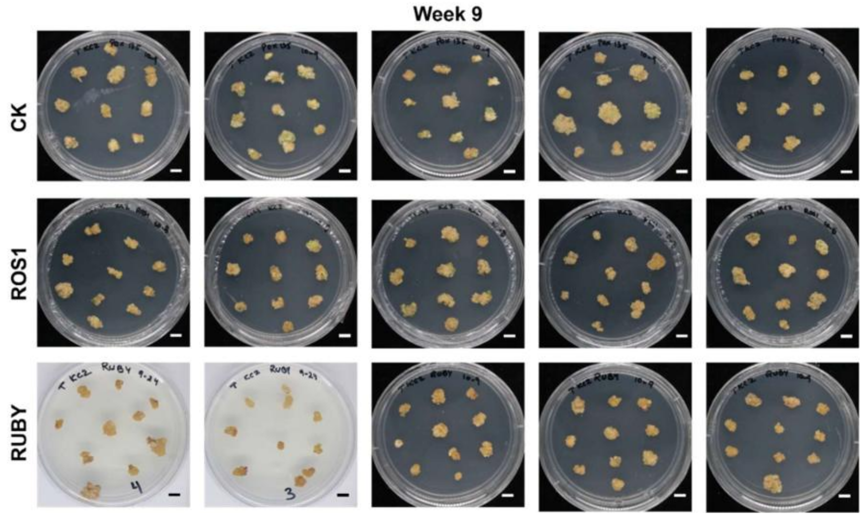

Supplementary Figure S4. Representative citrus explants during Agrobacterium-mediated transformation at week 9. Additional biological replicates of citrus explants under control (CK), ROSEA1 (ROS1), and RUBY treatments at week 9. Five representative plates per treatment are shown. Scale bars, 1 cm.

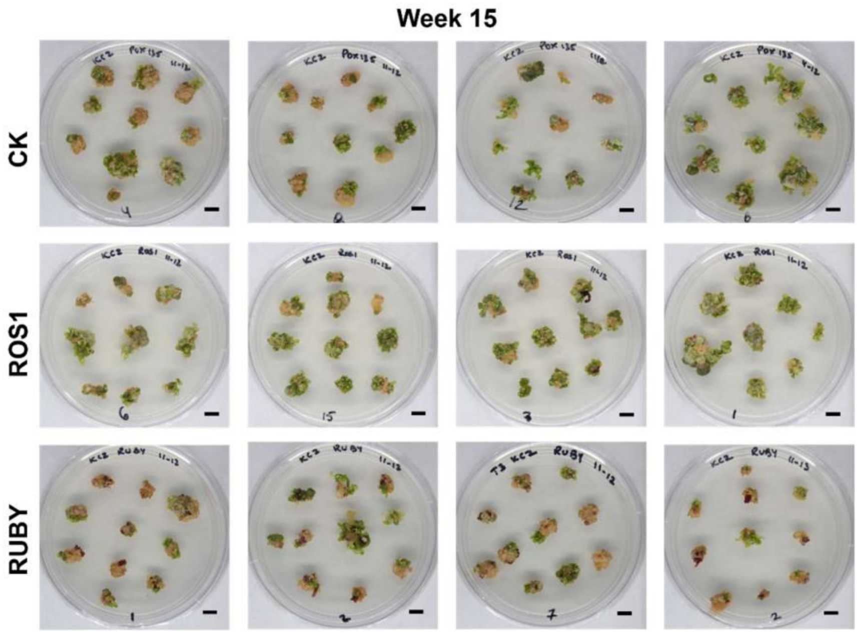

Supplementary Figure S5. Representative citrus explants during Agrobacterium-mediated transformation at week 15. Additional biological replicates of citrus explants under control (CK), ROSEA1 (ROS1), and RUBY treatments at week 15. Four representative plates per treatment are shown. Scale bars, 1 cm.

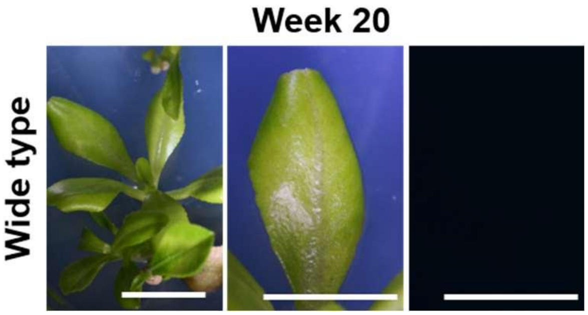

Supplementary Figure S6. Representative fully developed wild-type citrus plantlets at week 20. Whole-plant overview (left), close-up leaf images (middle), and GFP fluorescence (right) imaging of leaves are shown. Scale bars, 1 cm.

Description	Sequence	Amplicon size (bp)	Annotation
RUBY-F	CACGAACTCCAGCAGGACCATG	700	Transgenic plant genotyping PCR
RUBY-R	CTGGTCGAGCTGGACGGCGACG		
AmROSEA1-F	ATGGAAAAGAATTGTCGTGG	450	
AmROSEA1-R	TTAATTTCCAATTTGTTGGGC		
Insert-dec-F	atgaattCCAACATGGTGGAGCAC	CK: 1.1 kb; ROSEA1: 1.7 kb; RUBY: 3.2 kb	Plasmid diagnostic PCR
Insert-dec-R	TGCTGCAGAAGAGATCCAACA		

Supplementary Table S1. The list of primers used in this study.
